# The feeling of anger experienced by adults whose parents were alcohol addicts

**DOI:** 10.1192/j.eurpsy.2026.10863

**Published:** 2026-07-31

**Authors:** A. Lutsenko, A. Spivakovskaya

**Affiliations:** 1 Russian State University for the Humanities; 2 Moscow State University, Moscow, Russian Federation

## Abstract

**Introduction:**

Studies of individuals whose parents were alcohol addicts have shown an increased tendency toward addictive and suicidal behavior, difficulty establishing close relationships, and unclear boundaries of identity. Children living in situations of parental alcohol dependence often face an unstable family environment, which can lead to emotional suppression or excessive aggression. This can hinder the development of healthy interpersonal relationships and emotional intelligence in adulthood.

**Objectives:**

The aim of this study was to identify the characteristics of anger experienced by adults whose parents were alcohol addicts.

**Methods:**

The study involved 60 participants aged 18 to 45. The main study group consisted of 30 participants (18 women and 12 men) who were members of the “Adult Children of Alcoholics” community. They grew up and lived in families where their fathers abused alcohol and had received treatment for this disorder throughout their lives. The control group consisted of 30 participants of the “Adult Children of Alcoholics” community (15 men and 15 women) who grew up in families where their fathers suffered from alcohol addiction and who had experienced the death of their parents. We used: 1. Buss-Perry Aggression Questionnaire (BPAQ), 2. Rosenzweig Frustration Test, 3. Phenomenological analysis of interviews.

**Results:**

1) Individuals who grew up and live in alcohol families tend to experience anger more frequently and intensely than individuals who grew up in alcohol families and experienced parental death (U = 154 p <0,01). 2) Individuals who grew up and live in alcohol families experience difficulties self-regulating anger compared to individuals who grew up in alcohol families and experienced parental death ((U=286 p< 0,01). 3) Individuals who grew up and live in alcohol families often exhibit a higher tendency to use aggressive language in communication compared to those who grew up in alcohol families and experienced parental death.

**Conclusions:**

After the parents’ death, the experience of aggression is superimposed on the processes of experiencing the loss of a parent, as a result of which aggression can be transformed into the experience of guilt and a tendency to ruminate on past events; respondents blame themselves for the illnesses and deaths of their parents, strive to find evidence of their sinfulness and guilt in the present tense, often apologize and strive to make an emphatically pleasant impression on people.

**Disclosure of Interest:**

None Declared

